# ABCG1 contributes to suberin formation in *Arabidopsis thaliana* roots

**DOI:** 10.1038/s41598-019-47916-9

**Published:** 2019-08-06

**Authors:** Kalpana Shanmugarajah, Nicole Linka, Katharina Gräfe, Sander H. J. Smits, Andreas P. M. Weber, Jürgen Zeier, Lutz Schmitt

**Affiliations:** 1 Institute of Biochemistry, Heinrich-Heine University, Düsseldorf, Germany; 2 Institute of Plant Biochemistry, Heinrich-Heine University, Düsseldorf, Germany; 3 Institute for Molecular Ecophysiology of Plants, Heinrich-Heine University, Düsseldorf, Germany; 4 Cluster of Excellence on Plant Sciences (CEPLAS), Heinrich-Heine University, Düsseldorf, Germany

**Keywords:** Plant sciences, Biochemistry

## Abstract

Diffusion barriers enable plant survival under fluctuating environmental conditions. They control internal water potential and protect against biotic or abiotic stress factors. How these protective molecules are deposited to the extracellular environment is poorly understood. We here examined the role of the *Arabidopsis* ABC half-size transporter AtABCG1 in the formation of the extracellular root suberin layer. Quantitative analysis of extracellular long-chain fatty acids and aliphatic alcohols in the *atabcg1* mutants demonstrated altered root suberin composition, specifically a reduction in longer chain dicarboxylic acids, fatty alcohols and acids. Accordingly, the ATP-hydrolyzing activity of heterologous expressed and purified AtABCG1 was strongly stimulated by fatty alcohols (C_26_–C_30_) and fatty acids (C_24_–C_30_) in a chain length dependent manner. These results are a first indication for the function of AtABCG1 in the transport of longer chain aliphatic monomers from the cytoplasm to the apoplastic space during root suberin formation.

## Introduction

ATP binding cassette (ABC) transporters are ubiquitously expressed and able to transport diverse substrates across biological membranes^1^. Remarkably, plant genomes encode a large number of ABC proteins, with for example 130 genes in *Arabidopsis thaliana* that are classified into eight subfamilies^2,3^. The ABCG subfamily forms the largest subfamily, with 28 half-size (white brown complex, WBC) and 15 full-size (pleiotropic drug resistance, PDR) transporters. The characteristic feature of the ABCG subfamily is their inverse domain organization where the NBDs are fused N-terminally to the TMDs. Further, WBC proteins only obtain their functional state after dimerization^3^.

Half-size ABCGs in *A. thaliana* have been found to be involved in antibiotic resistance^4,5^, ABA, and cytokinin transport^6–11^, or stomatal regulation in guard cells^12,13^.

Notably, the majority of the analyzed half-size AtABCGs have been shown to be part of diffusion barrier formation. Diffusion barriers, such as cutin or suberin, which are deposited in the cell wall, enable plants to prevent water loss or serve as protective layers against many environmental stress factors, including drought and pathogen attack^14^. Several half-size AtABCG transporters have been shown to be involved in pollen protection. Specifically, AtABCG26 has been shown to be important for exine formation. A double mutant defective in AtABCG1 and AtABCG16 indicated a role in pollen nexine and intine layer formation, as well as in post-meiotic pollen development^15–21^. Additionally, AtABCG9 acts together with the full-size transporter AtABCG31 in pollen coat maturation^22^. In some cases, ABCG half-size transporters from the same phylogenetic clade act together in order to build up specific diffusion barriers. For example, AtABCG11, AtABCG12, and AtABCG13 are involved in cuticle formation^2,23–28^. Another clade is formed by AtABCG1, AtABCG16, AtABCG2, AtABCG6, and AtABCG20. Amongst these, all clade members except for AtABCG1 and AtABCG16 were reported to be part of suberin formation in *Arabidopsis* roots and seeds^2,21,29^.

The suberin polymer consists of an aliphatic polyester, which is linked with phenolic components and associated with waxes. Typical monomers of the aliphatic suberin domain are ω-hydroxy acids, α,ω-dicarboxylic acids, fatty acids and alcohols. Here, glycerol is esterified to the ω-hydroxy and α,ω-dicarboxylic acids and the phenylpropanoid pathway derived phenolic compounds^30,31^. The suberin monomers are synthesized in specific cell compartments and transported through the plasma membrane for final assembly into the suberin polymer in the apoplast. Current hypotheses for suberin precursor transport to the cell wall include transport via the secretory pathway, passive transport of oleophilic bodies, or transport by ABC transporters^32,33^. Recently, a sequence comparison of all AtABCGs with a potential suberin precursor transporter from *Solanum tuberosum* (StABCG1), revealed that AtABCG1 and AtABCG16 share the highest sequence identity^34^.

This study combines *atabcg1* mutant analysis and functional assays with heterologous overexpressed and purified AtABCG1 to reveal the role of the AtABCG1 transporter in root suberin formation. Both complementary assays indicate that AtABCG1 transports specific fatty acids and fatty alcohols precursors of the suberin synthesis and thus contributes to suberin barrier formation in the roots.

## Methods

### Phenotypic analysis of Arabidopsis T-DNA insertion lines

#### Plant material and growth conditions

*Arabidopsis* wildtype plants (ecotype Columbia (Col-0)) and the *Arabidopsis* SAIL T-DNA line SAIL563B03 (*atabcg1-3*) were obtained from the Nottingham Arabidopsis Stock Centre (NASC) (www.arabidopsis.info). The *Arabidopsis* GABI-Kat T-DNA line GK850E07 (*atabcg1-4*) was ordered via GABI-Kat (www.gabi-kat.de). Wildtype and mutant seeds were surface-sterilized using a vapor-sterilization method^35^. Seeds were stratified for at least 3 days and germinated on half-strength MS medium with 0.8% (w/v) plant agar. Plants were transferred to soil after 14 days and grown under long day conditions (16 h light (100 μmol m^−2^ s^−1^)/8 h darkness) at 22 °C (day)/18 °C (night).

#### Identification of homozygous T-DNA insertion lines

At least 4-week-old soil-grown wildtype and AtABCG1 T-DNA insertion lines were used to identify plants homozygous for the T-DNA insertion. The genomic DNA was isolated using the DNeasy Plant Mini Kit (Qiagen) and genotyped by standard PCR reactions with gene-specific and T-DNA/gene junction specific primer pairs (Table S1). The actin gene ACT2 (At3g18780) was amplified as positive control with primer pairs P67/P68. Independent homozygous *atabcg1* mutant plant were further propagated.

#### RT-PCR and qRT-PCR

Total RNA was isolated from 3-weeks-old MS-agar grown wild type and *atabcg1-3* and *atabcg1-4* mutant seedlings using the RNeasy Plant Mini Kit (Qiagen). Samples were subjected after DNase treatment (RQ1 RNase-free DNase, Promega) to cDNA synthesis via Luna Script RT-PCR (NEB Biolabs) and analyzed for presence of full-length AtABCG1. Subsequent relative quantification of the *AtABCG1* gene in wildtype and mutant lines was performed using SYBR green-based PCR assay Luna Universal qRT-PCR (NEB Biolabs). The primer pairs QP1/QP2, QP3/QP4 and QP7/QP8 were used for gene-specific amplification (Table S1). The *AtABCG1* expression levels were to normalized against those of the Type 2A phosphatase interacting protein 41 (TIP41)-like reference gene (At4g34270) called Black^36^. Transcript level was calculated based on ∆CT values compared to the wild type^37,38^.

#### Gas chromatography analysis of AtABCG1 T-DNA insertion lines

Quantitative suberin analysis was performed with 10-week-old soil grown plants (adapted to^39,40^). Roots were carefully removed from the potting mixture and washed with deionized water to remove contaminants, cut into 5 mm pieces and dried to constant mass at 60 °C. 4 mg samples were first extracted with 1 ml chloroform/methanol/water (1:2.5:1; v/v), incubated for 1 h at 50 °C and washed twice with 1 ml methanol. After adding 5 µg of the internal standards heptadecanoic acid, 1-pentadecanol, and 15-hydroxypentadecanoic acid, suberin was depolymerized by transesterification in 1 ml boron trifluoride in methanol (10%, Fluka) for 24 h at 70 °C. The methanol lysate was washed twice with 1.5 ml dichloromethane and 1 ml saturated sodium chloride, respectively. The fused organic phases were subjected to solvent extraction with Na_2_SO_4_ and extensively dried under inert N_2_ gas. Subsequently, 16 µl of BSTFA (*N*, *O*-Bis(trimethylsilyl)trifluoroacetamide) and pyridine, respectively, and 80 µl of dichloromethane were added. Samples were incubated at 70 °C for 30 min before they were analyzed by GC-MS (Agilent Technologies 7890A gas chromatograph coupled to a 5975C mass spectrometric detector; GC equipped with a HP-1MS column, J & W Scientific) in the electron ionization (EI) mode. The GC temperature program was as follows: initial temperature 50 °C for 2 min, gradient to 150 °C at 10 °C/min, 1 min hold at 150 °C, gradient to 290 °C at 5 °C/min, gradient to 320 °C at 20 °C/min, final hold at 320 °C for 5 min. Suberin compounds were quantified by integration of specific ion chromatograms. Thereby, a correction factor of 1 was assumed and quantifier ions and retention times were used from Table S2. Heptadecanoic acid was used as internal standard for the quantification of p-coumaric acid, ferulic acid, α, ω-dicarboxylic acids and fatty acids, 15-hydroxypentadecanoic acid for ω-hydroxyacids and 2-hydroxyacids, and 1-pentadecanol for fatty alcohols.

### *In vitro* characterization of AtABCG1

#### Native PAGE of AtABCG1

The native oligomeric state of AtABCG1 was determined by native gel electrophoresis using a 4–16% Native PAGE Bis-Tris Gel (Invitrogen). Protein bands were visualized by Coomassie brilliant blue staining.

#### Cloning of AtABCG1 for expression in *P. pastoris*

As previously described, a synthetic gene of *AtABCG1* (At2g39350) with codon optimization for expression in *P. pastoris* (Invitrogen) was cloned by In-Fusion into the expression vector pSGP18-Ntag^41^. An ATPase hydrolysis deficient *AtABCG1* mutant was generated by site-directed mutagenesis. First Glu259 in the conserved Walker B motif was replaced by Gln with the primer pairs ABCG1-EQ fwd/rev and additionally His291 of the H-loop was replaced by Ala with the primer pairs ABCG1-HA fwd/rev (Table S1). Sequences were verified by DNA sequencing (GATC Biotech).

#### Transformation and expression of AtABCG1 in *P. pastoris*

The expression constructs pSGP18-AtABCG1-Ntag and pSGP18-AtABCG1-EQ/HA-Ntag were transformed into *P. pastoris* cells as described in [42]. Briefly, 20 µg of the respective plasmid DNA was linearized with *Mss*I (Thermo Fisher Scientific) and transformed into 80 µl electro-competent X33 cells (Life Technologies). Subsequently, clones were selected on YPD-agar supplemented with 500 µg/ml zeocin and analyzed for expression^42^. Positive clones were fermented in a 15L table-top glass fermenter (Applikon Biotechnology) according to the Invitrogen *Pichia* fermentation guidelines.

#### Isolation of AtABCG1 crude membranes

100 g wet cells of AtABCG1 expressing *P. pastoris* cells were thawed on ice and re-suspended with lysis buffer (50 mM Tris-HCl, pH 7.5, 150 mM NaCl, 1 mM EDTA, 1 mM EGTA, 0.3M sucrose) and protein inhibitor cocktail (Roche) was added to a final concentration of 0.5 g cells/ml. The cells were disrupted by two passages through a Cell Disruptor (Constant System) at 2.5 kbar and the cell lysate was centrifuged twice (15, 000 × g, 30 min, 4 °C). Subsequently, the supernatant was centrifuged for 1 h at 125, 000 × g, 4 °C. Crude membranes were re-suspended in buffer A (50 mM Tris-HCl, pH 8, 150 mM NaCl, 15% glycerol), flash frozen in liquid N_2_ and stored at −80 °C until further usage.

#### Solubilization screen via dot blot technique

AtABCG1 containing crude membranes were thawed on ice and solubilized in 200 μl buffer A. While the membrane concentration was adjusted to 5 mg/ml, detergents were used at 1% (w/v) or higher depending on their critical micellar concentration (cmc). A list of detergents used is provided in the Supplementary Information (Table S3). The solubilization was carried out for 1 hour at 4 °C on a rotator, afterwards samples were centrifuged (100,000 × g, 30 min, 4 °C) and the supernatant supplemented with SDS sample buffer. After heating (65 °C, 10 min) 3 μl of the respective samples were spotted on a nitrocellulose membrane and dried completely. The membrane was blocked for 1 h in TBS-T with 5% (w/v) milk powder and the target protein analyzed by immunoblotting (anti-His-tag antibody).

#### Purification of AtABCG1

The purification of AtABCG1 and AtABCG1-EQ/HA was conducted by calmodulin binding peptide affinity (CBP) chromatography. The crude membrane concentration, equivalent to 50 g wet cells, was adjusted with buffer A to 5 mg/ml. Subsequently, membranes were solubilized with 1.5% Fos Choline-14 (Anatrace) for 2 hours at 4 °C and centrifuged (125, 000 × g, 1 h, 4 °C). The supernatant was diluted 3-times with CBP binding buffer (50 mM Tris-HCl, pH 8, 10 mM CaCl_2_ 150 mM NaCl, 1 mM Magnesiumchlorid,10% Glycerin) and 2× cmc Fos-Choline-14 and 2 ml CBP resin (Agilent Technologies, Santa Clara, CA) were incubated and the sample was incubated over night at 4 °C on a rotator. The resin was transferred to a gravity flow column (Bio-Rad), washed with CBP binding buffer and eluted with CBP elution buffer (50 mM Tris-HCl, pH 8, 150 mM NaCl, 2 mM EGTA, 10% Glycerin) in the presence of 2× cmc Fos-Choline-14. The purified protein was immediately used for ATPase activity assays or aliquoted, snap frozen in liquid nitrogen and stored at – 80 °C. For Blue Native PAGE (BN-PAGE), proteins were concentrated with a 100 kDa cutoff protein concentrator (Thermo Scientific™ Pierce™) to their final concentration. Samples were analyzed after heating (65 °C, 10 min) by Colloidal Coomassie stained SDS-PAGE and immunoblotting.

#### ATPase activity measurements of AtABCG1

The malachite green assay was performed with the following adaptations as outlined by Baykov *et al*.^43^. Reactions were performed in a total volume of 25 µl in reaction buffer containing 10 mM MgCl_2_. The reaction was started after adding 0.3–2 µg of the purified protein together with 5 mM ATP, incubated at 25 °C and quenched after 40 min by transfer into 175 µl 20 mM H_2_SO_4_. After adding 50 µl dye solution (0.096% (w/v) malachite green, 1.48% (w/v) ammonium molybdate, and 0.173% (w/v) Tween-20 in 2.36M H_2_ SO_4_) samples were incubated for 10 min at room temperature and the amount of free inorganic phosphate (P_i_) was quantified thereafter by measuring the absorption at 595 nm. Samples without 10 mM MgCl_2_ were used to subtract background values and a Na_2_HPO_4_ standard curve for free phosphate calibration. For substrate stimulated ATPase activity, substrates were dissolved in chloroform or chloroform/methanol (1:1; v/v) respectively. Therefore, additional control reactions were performed in the presence of the respective solvents. Kinetic parameters were determined using different ATP concentrations ranging from 0 to 8 mM. The controls were subtracted for statistical analysis. Kinetic data were fitted using the GraphPad Prism 7 Software according to the Michaelis-Menten equation:

$$v=\frac{{v}_{\max }[S]}{{K}_{m}+[S]}$$

here, v is equivalent to the ATPase activity, v_max_ the maximal ATPase activity, S the substrate concentration and K_m_ the Michaelis-Menten constant.

#### Sequence alignments

Protein alignments of AtABCG1 (At2g39350); ACT2 (At3g18780); StABCG1, (XP_006345915) and PhABCG1 (JQ088099) were performed using the CLUSTALW program (www.ebi.ac.uk/Tools/clustalw) with default settings.

## Results

### Effect of AtABCG1 knock-out on *Arabidopsis* root suberin composition

In order to infer the physiological role of AtABCG1, sequence comparison with selected ABCG1 transporters from other plants was performed (Fig. S1). While StABCG1 is involved in suberin formation, PhABCG1 (*Petunia hybrida*) has been reported to mediate volatile transport^34,44^. AtABCG1 shares with 74%, the highest amino acid identity with StABCG1 and only 31% with PhABCG1. Separate NBD and TMD alignments exhibited a similar result with 69% and 81% identity for StABCG1 and 33% and 26% for PhABCG1.

**Figure 1:**
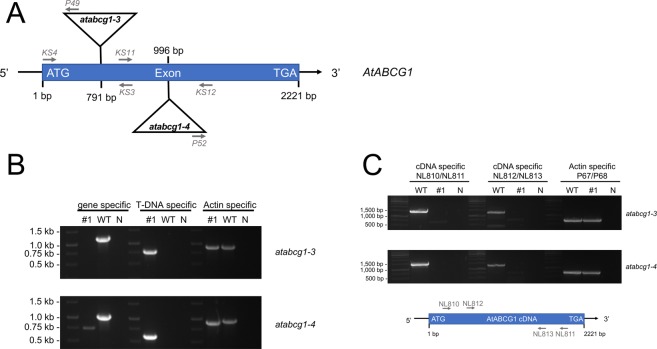
Characterization of T-DNA insertional *atabcg1* mutant lines. (**A**) Genomic organization of the two independent T-DNA insertional *atabcg1* mutant lines, *atabcg1-3* and *atabcg1-4*. The *atabcg1-3* line contains a T-DNA insertion at 791 bp and the *atabcg1-4* line one at 996 bp. Arrows indicate the primers used for the mutant characterization. (**B**) Genetic analysis of the *atabcg1* mutant lines *atabcg1-3* and *atabcg1-4*. Genomic DNA of wildtype (WT) and mutant lines (#1) were analyzed for *AtABCG1* gene specific, T-DNA specific and actin specific amplification products. N, negative control. (**C**) Presence of full-length AtABCG1 transcripts in T-DNA insertional *atabcg1* mutant lines. Amplification of full-length AtABCG1 was performed from cDNA isolated from *atabcg1-3* and *atabcg1-4* lines and the depicted primer pairs. Entire gels can be found in supplemental information (Figs S5 and S6).

To investigate the role of AtABCG1 in root suberin formation, two independent T-DNA *A. thaliana* insertional mutant lines in the Col-0 background, *atabcg1-3* (SAIL563B03) and *atabcg1-4* (GK850E07), were obtained from Nottingham Arabidopsis Stock Center and GABI-Kat, respectively (Fig. 1A). Homozygous *atabcg1-3* and *atabcg1-4* plants were identified by genomic PCR (Fig. 1B). Both homozygous mutant plants were verified as AtABCG1 knock-out lines, since the full-length *AtABCG1* transcript was absent in *atabcg1-3* and *atabcg1-4* (Fig. 1C).

**Figure 2:**
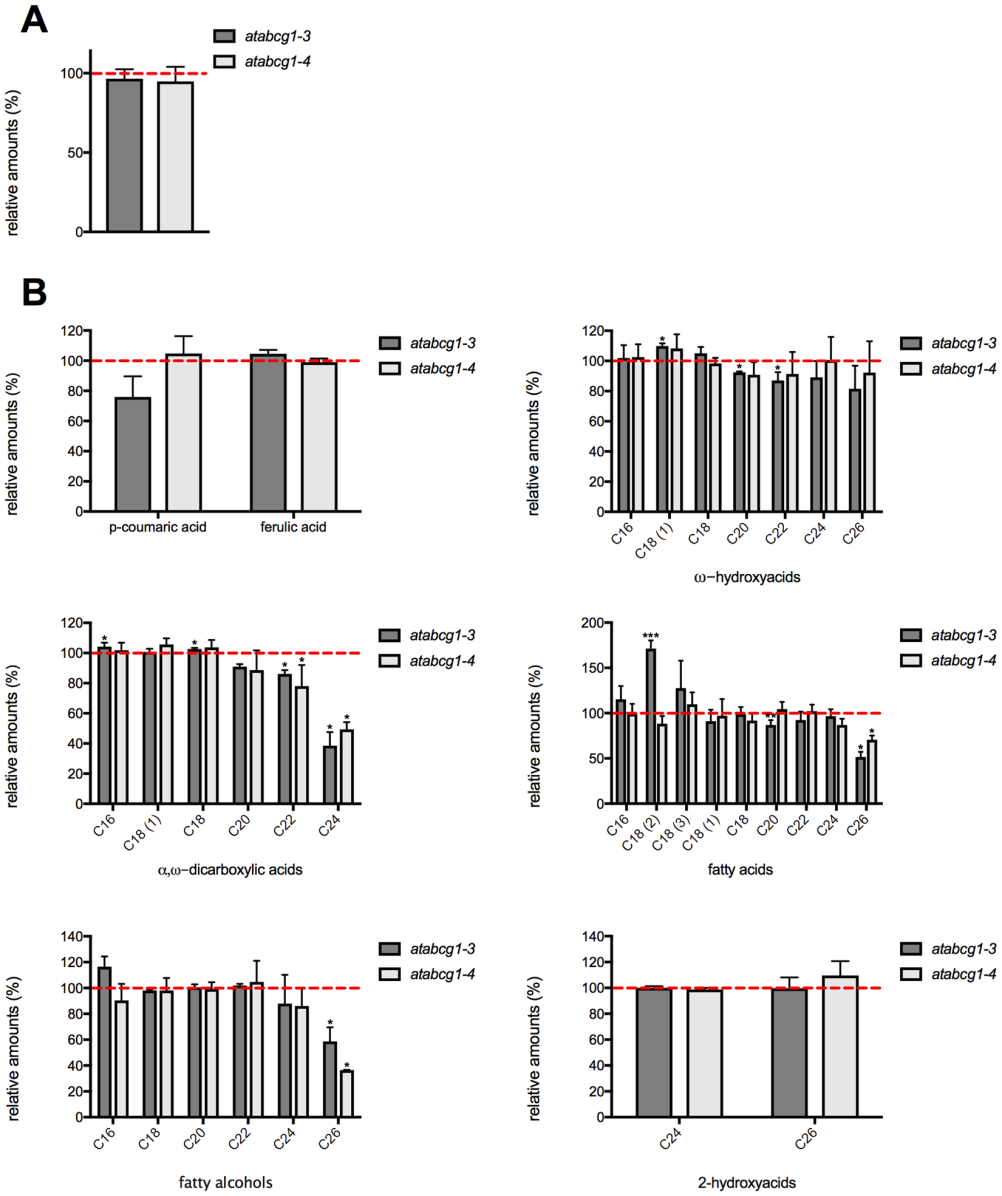
Suberin composition in *Arabidopsis* wildtype and *atabcg1* mutant plants. (**A**) Suberin content in wildtype and *atabcg1* mutant lines. Relative amount of released suberin after transesterification of wildtype and T-DNA insertional *atabcg1* mutant lines *atabcg1-3* and *atabcg1-4*. Data represent mean ± SD of at least 4 independent experiments. Wildtype (n = 7), *atabcg1-3* (n = 4) and *atabcg1-4* (n = 5) independent experiments. (**B**) Monomer composition of *Arabidopsis* wildtype and *atabcg1* mutant root suberin. The relative abundance of suberin monomers in the independent T-DNA insertional mutant lines *atabcg1-3* (n = 4) and *atabcg1-*4 (n = 4) per substance class is depicted in comparison to the wildtype (n = 4). The suberin monomers were quantified using quantifier ions and retention times from Supplemental Table S2. The red lines indicate the relative abundance of the respective monomers in the wildtype (set to 100%). Data represents mean and ±SD. Significance analysis between *atabcg1* mutants and wildtype samples were performed by t test. (two-tailed): ***P ≤ 0.0001, **P ≤ 0.01, *P ≤ 0.05.

Subsequently, roots from 10-week-old soil-grown wild-type and two independent *atabcg1* mutant lines were analyzed for suberin formation to infer a possible role of AtABCG1 in the *Arabidopsis* root. Suberin monomers were released from the respective root sample by transesterification and their relative abundance determined by GC-MS analysis. The relative amount of the identified suberin monomers was observed to be similar in both wildtype and *atabcg1* mutant lines (Fig. 2). Qualitatively, the de-esterifiable part of the root suberin of both wildtype and *atabcg1* mutant was composed of ω-hydroxycarboxylic acids with carbon chain length ranging from C_16_ to C_26_, α,ω-dicarboxylic acids (C_16_ to C_24_), fatty acids (C_16_ to C_26_), fatty alcohols (C_16_ to C_26_), and cinnamic acids (p-coumaric acid and ferulic acid) (Fig. 2B). The chain length distribution of each substrate class was essentially identical to that of Franke *et al*., except that we detected small amount of C24 and C26 compounds^45^. Notably, the contents of several α,ω-dicarboxylic acid, fatty acid, and fatty alcohol monomers with higher chain lengths were significantly lower in the suberin of the *atabcg1* mutant plants than in the suberin of the wildtype (Fig. 2B). Compared to the wildtype, the relative amounts of the α,ω dicarboxylic acids C_22_ and C_24_ showed a chain-length depending decrease to around 80% to 50% in *atabcg1-3* and *atacbg1-4*, whereby the strongest reduction was observed for C_24_ α,ω dicarboxylic acid (38.5 ± 9.0% for *atabcg1-3*; 49.4 ± 4.8% for *atacbg1-4*). The relative amounts of C_24_ fatty acids (96.6 ± 7.6% for *atabcg1-3*; 86.9 ± 6.8% for *atacbg1-4*) and fatty alcohols (87.8% ± 22.3 for *atabcg1-3*; 86.1 ± 13.6% for *atacbg1-4*) were not reduced. By contrast, the relative amounts of C_26_ fatty acids and fatty alcohols were reduced to 60% (51.5 ± 5.7% for *atabcg1-3*, 70.6 ± 4.7% for *atacbg1-4*) and 50% (58.6 ± 11.0% for *atacbg1-3*; 36.4 ± 0.2% for *atacbg1-4*) of wildtype values. Finally, the relative amounts of C_16_, C_18_, C_20_ and C_22_ monomers were generally similar in the suberin of wildtype and *atacbg1* mutants for all of the substance classes (Fig. 2B).

Thus, the analysis of suberin composition in *atabcg1* mutants provides an indication for the involvement of AtABCG1 in root suberin composition. However, direct functional assays are essential for unambiguous substrate identification. Consequently, we heterologously expressed and purified AtABCG1 for further functional characterization.

### Cloning, expression and purification of AtABCG1

**Figure 3:**
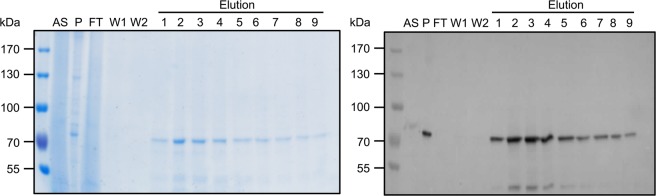
Calmodulin binding peptide (CBP) purification of AtABCG1. AtABCG1 was solubilized in Fos Choline-14 and purified by CBP resin. Fractions were analyzed after 10 min incubation at 65 °C via 7% SDS-PAGE (left panel) or immunoblotting (anti-His-tag antibody, right panel). AtABCG1 has a calculated molecular weight of 86 kDa. The lower 45 kDa band in the elution fractions 2–6 arises as verified by immunoblotting also from AtABCG1 and is likely a degradation product. Pre-stained molecular weight markers are shown on the left. AS, after solubilization; P, pellet; FT, flow through; W1-W2, washing fraction. The colors of the immunoblot were inverted without contrast changing.

AtABCG1 and AtABCG1-EQ/HA were successfully cloned and overexpressed in the methylotrophic yeast *P. pastoris*. An ATP hydrolysis deficient *AtABCG1-EQ/HA* mutant was generated as a negative control by the implementation of one mutation (E259Q) in the Walker B motif and another mutation (H291A) in the H-loop of the NBD^46,47^. Expression and purification of *AtABCG1* and *AtABCG1-EQ/HA* was performed using identical protocols. In order to determine a suitable detergent for solubilization of AtABCG1, we screened 95 detergents (Fig. S3 and Table S3) by the dot-blot technique. Fos-Choline-14 was found as a suitable detergent to efficiently solubilize AtABCG1 from the *Pichia* membranes^42^. For large scale purification, the isolated *Pichia* membranes were adjusted to 5 mg/ml and solubilized with 1.5% (w/v) Fos-Choline-14 for 2 hours at 4 °C. Subsequently, solubilized AtABCG1 was purified using calmodulin binding-peptide (CBP) chromatography via the CBP-tag introduced genetically at the N-terminus yielding approximately 2 mg AtABCG1 per 100 g wet cells. Purified fractions were analyzed with respect to purity via Colloidal Coomassie stained SDS-PAGE and identified by immunoblotting using an anti-His antibody (Fig. 3). This further revealed that AtABCG1 was purified to homogeneity as judged by the SDS-PAGE.

### AtABCG1 can homodimerize

**Figure 4:**
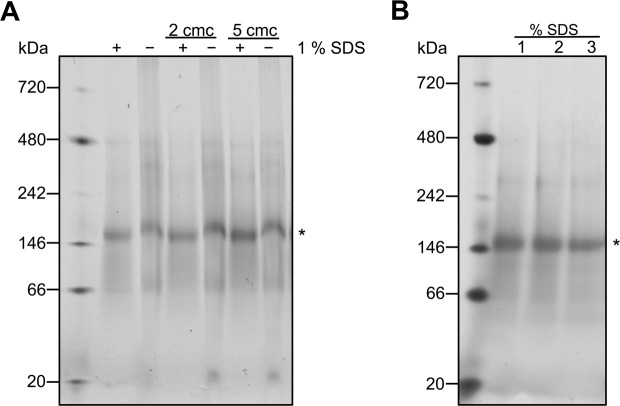
Native oligomeric state of AtABCG1. CBP purified AtABCG1 was subjected to 4–16% Bis-Tris BN-PAGE. (**A**) Samples were analyzed in different Fos Choline-14 concentration (0× cmc, 2× cmc, 5× cmc). Native AtABCG1 partially migrates under SDS substitution mainly with 172 kDa as homodimeric (*) protein. (**B**) Samples were submitted with 2× cmc Fos-Choline 14 and analyzed in different SDS-concentration (1%, 2%, 3%). A molecular weight marker is shown in the left.+, samples substituted with 1% SDS; -, samples without SDS. The colors of the immunoblot were inverted without contrast changing.

Since AtABCG1 is a half-size transporter, the gene encodes a single NBD and a single TMD. Half-size transporters have to homo- or heterodimerize in order to form a functional transporter^3^. CBP-purified AtABCG1 migrates on SDS-PAGE as monomer with an approximate molecular weight of 86 kDa (Fig. 3). The corresponding BN-PAGE (Fig. 4) demonstrated that the native oligomeric state of AtABCG1 was dimeric and the oligomerization was not reversed by addition of 1% SDS. A complete monomerization was also not observed even in the presence of 3% SDS, which indicates that AtABCG1 is a very stable homodimer. Hence AtABCG1 is mainly present as a homodimer and further protein-protein interaction studies might reveal potential interaction partners that would results in heterodimerization as described for AtABCG11 and AtABCG12^48^.

### ATPase activity of purified AtABCG1

**Figure 5:**
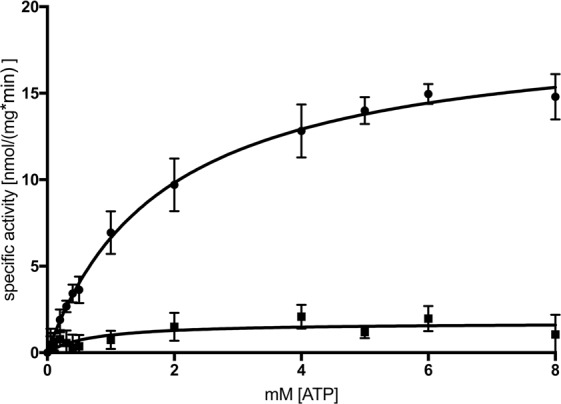
ATPase activity of purified AtABCG1 and AtABCG1-EQ/HA mutant. The ATPase activity of CBP purified AtABCG1 (circles) and the EQ/HA mutant (squares) was measured in presence of 0 to 8 mM ATP. Data was analyzed according to Michaelis-Menten equation and represents mean and ±SD of three independent replicates.

We used a malachite green based ATPase assay^43^ to analyze the specific activity of detergent-purified AtABCG1. Background activity from impurities was determined by the ATPase hydrolysis deficient mutant AtABCG1-EQ/HA, which was purified in the exact same way yielding a sample of similar purity and subtracted prior to analysis. The concentration of purified AtABCG1 and AtABCG1-EQ/HA was kept constant in the assay, while ATP concentration was varied from 0 mM to 8 mM. Subsequently, data analysis was performed according to the Michaelis-Menten equation (see Materials and Methods). Wildtype AtABCG1 exhibited a maximal reaction velocity v_max_ of 17.1 ± 0.1 nmol min^−1^ per mg purified AtABCG1 and a K_m_ value of 1.8 ± 0.1 mM (Fig. 5, Table 1).

**Table 1:** Kinetic parameters of AtABCG1 and AtABCG1-EQ/HA.

Protein	Km [mM]	v_max_ [nmol/(mg*min)]
AtABCG1	1.8 ± 0.1	18.8 ± 0.4
AtABCG1-EQ/HA	0.7 ± 0.4	1.7 ± 0.3

### Substrate stimulated ATPase activity of AtABCG1

**Figure 6:**
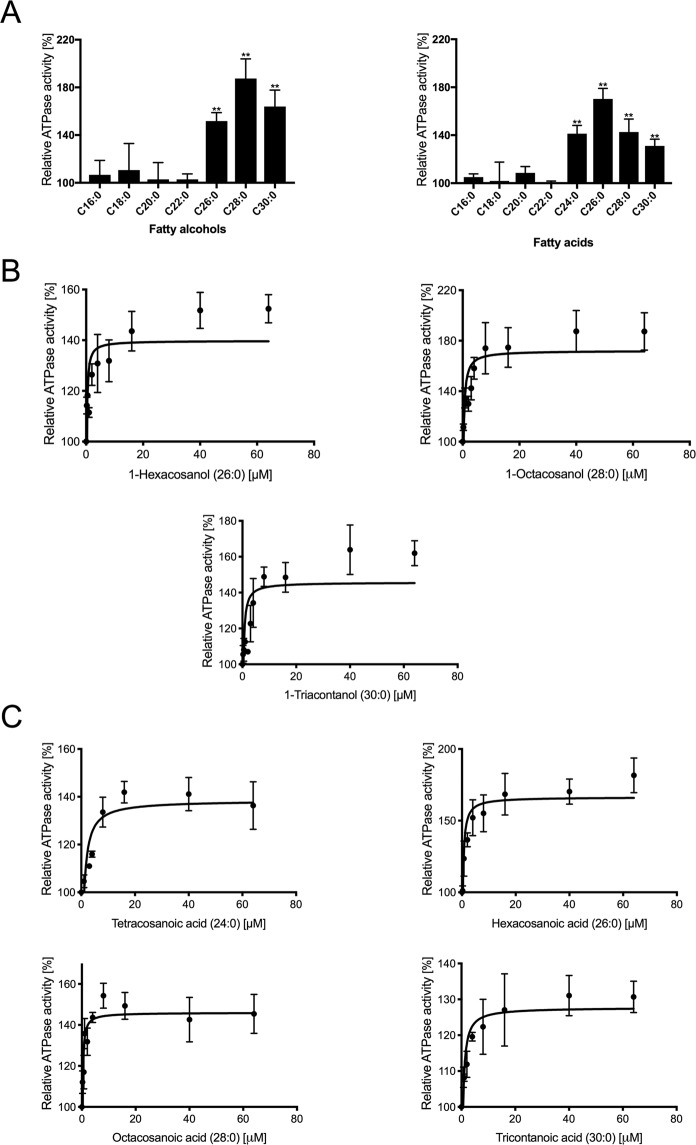
Substrate stimulated AtABCG1 ATPase activity. (**A**) Relative ATPase activity of AtABCG1 in presence of 40 µM fatty acids and fatty alcohols (C_16_–C_30_). Significance analysis between basal activity and substrate stimulated ATPase activity was performed by t test. (two-tailed): ***P ≤ 0.0001, **P ≤ 0.01, *P ≤ 0.05. (**B**,**C**) Concentration dependent relative AtABCG1 activity in the presence of fatty acids (C_24_–C_30_) and fatty alcohols (C_26_–C_30_). The ATPase activity of the EQ/HA mutant was subtracted and data fitted according to Michaelis-Menten kinetics. Data represent mean and ±SD of at least three independent replicates. An ATPase activity of 100% corresponds to the basale activity of AtABCG1 in the absence of any substrate.

Based on the above reported amino acid sequence comparison of AtABCG1 with StABCG1 and PhABCG1, and the *atabcg1* knockout studies performed, functional assays with purified AtABCG1 and potential substrates were performed. We examined, whether the specific activity of AtABCG1 was affected by potential PhABCG1 substrates, since AtABCG1 is also highly expressed in the flowers^21^. The AtABCG1 specific ATP hydrolysis was however not changed in presence of the volatile methyl benzoate, which is a reported PhABCG1 substrate^44^. The analysis of another potential PhABCG1 substrate, benzyl alcohol, was unfortunately not possible in our assay due to its viscous character. Subsequently performed ATPase activity measurements in the presence of suberin precursor molecules (Fig. S4), which were commercially available, verified the above obtained results of the *atabcg1* mutant studies. Thereby, fatty alcohols and acids with chain length of C16 to C30 were analyzed and a significant effect was seen for C26 to C30 and C24 to C30, respectively (Fig. 6A). The effect of 1-tetracosanol (C24) was not analyzed as it precipitated under the applied assay conditions.

As summarized in Fig. 6B,C, further assays with varying substrate concentration ranging from 0 µM to 65 µM revealed stimulatory effects according to Michael-Menten equation suggesting that these compounds represent substrates of AtABCG1. In detail, ATPase activity was stimulated by 1-hexacosanol (v_max_ 50.7 ± 4.0%), 1-octacosanol (v_max_ 90.1 ± 4.8%), 1-triacontanol (v_max_ 69.6 ± 5.1%), tetracosanoic acid (v_max_ 46.1 ± 5.2%), hexacosanoic acid (v_max_ 78.5 ± 3.3%), octacosanoic acid (v_max_ 49.5 ± 3.2%) and tricontanoic acid (v_max_ 32.3 ± 0.8%) (Table 2). Thereby, 1-triacontanol and tetracosanoic acid displayed a K_m_ value with 5.3 ± 1.2 µM and 5.4 ± 2.1 µM, respectively. A correlation between kinetic values and chain length was only partially observed. The stimulatory effect increased with the substrate chain length up to 1-octocosanol (C_28_) and hexacosanoic acid (C_26_), respectively and decreased thereafter. In consistence, phenotypical studies of AtABCG1 lacking plants demonstrated a decreased relative abundance of C_26_ fatty acid (hexacosanoic acid) (51.5 ± 5.7% for *atabcg1-3*, 70.6 ± 4.7% for *atacbg1-4*) (Fig. 2). While the *atabcg1* mutant plants contained still 90% relative abundance of C_24_ fatty acid (tetracosanoic acid) (96.6 ± 7.6% for *atabcg1-3*; 86.9 ± 6.8% for *atacbg1-4*), was the specific activity of purified AtABCG1 stimulated up to up to 50% (46.1 ± 5.2%). A further compliance was found for the C_26_ fatty alcohol (1-hexacosanol), which was significantly reduced (58.6 ± 11.0% for *atacbg1-3*; 36.4 ± 0.2% for *atacbg1-4*) in AtABCG1 lacking plants and stimulated the ATPase activity of AtABCG1 up to 50% (50.7 ± 4.0%).

**Table 2:** AtABCG1 stimulation in presence of fatty alcohols and fatty acids.

Substrate	Km [µM]	v_max_ [%]
***Fatty alcohols***		
1-Hexacosanol (26:0)	2.1 ± 0.7	50.7 ± 4.0
1-Octacosanol (28:0)	2.4 ± 0.4	90.1 ± 4.8
1-Triacontanol (30:0)	5.3 ± 1.2	69.6 ± 5.1
***Fatty acids***		
Tetracosanoic acid (24:0)	5.4 ± 2.1	46.1 ± 5.2
Hexacosanoic acid (26:0)	2.3 ± 0.4	78.5 ± 3.3
Octacosanoic acid (28:0)	0.7 ± 0.2	49.5 ± 3.2
Tricontanoic acid (30:0)	2.9 ± 0.2	32.3 ± 0.8

Thus, direct functional assays with purified AtABCG1 revealed C_26_–C_30_ fatty alcohols and C_24_–C_30_ fatty acids as potential AtABCG1 substrates, whereas suberin analysis of both *atabcg1* mutants indicated C_24_ and C_26_ fatty acids and C_26_ fatty alcohol as AtABCG1 substrates. According to the root suberin analyses, C_22_ and C_24_ α,ω dicarboxylic acids also act as putative AtABCG1 substrates. However, subsequent direct functional assays could not be performed as these compounds were not commercially available. Nevertheless, both root suberin analyses and biochemical assays coincidingly suggest that aliphatic suberin monomers with higher chain length (C_24_ or C_26_) function as *in vivo* substrates of AtABCG1 in *Arabidopsis* roots.

## Discussion

Suberin is a glycerolipid polymer, that forms particularly in liaison with the Casparian strips the initial barrier for the movement of water and apoplastic solutes in the roots^14,33,49^. Several half-size transporters across different plant species have been shown to be involved in suberin barrier formation^21,29,34,50^. In *Arabidopsis* ABCG2, ABCG6, ABCG20 and ABCG11 were found to be involved in suberin precursor transport^21,26,29^. The half-size transporter AtABCG1 originates from the same phylogenetic subclade as AtABCG2, AtABCG6 and AtABCG20^21,29^. Moreover, sequence comparison of AtABCG1 with StABCG1 and PhABCG1, homonymous half-size transporters that are supposed to transport suberin precursors and volatiles, respectively, revealed the highest identity amongst AtABCG1 and StABCG1 (with 74% sequence, 69% NBD sequence and 81% TMD sequence identity). Therefore, the present study aimed to advance the knowledge on the physiological role of the half-size transporter AtABCG1, particularly in suberin formation. First, phenotypic analysis of T-DNA insertional *atabcg1* mutants demonstrated a changed root suberin composition. Nevertheless, since a gene knock-out could also impact for instance related pathways or transporters and thus lead to artefactual results, direct functional assays are essential for unambiguous substrate identification^51^. Therefore, AtABCG1 was heterologously expressed in *P. pastoris* and detergent purified. Subsequent Blue native PAGE analysis demonstrated that Fos-Choline-14 solubilized AtABCG1 is a homodimer. Similarly, another half-size transporter AtABCG11 adopts also a homodimeric state^48^, but it was found to form heterodimers with AtABCG12, AtABCG9 and AtABCG14^9,48^. Hence, protein-protein interaction studies might reveal potential interaction partners and further physiological roles of AtABCG1. However, we were able to confirm the enzymatic activity of purified AtABCG1, as briefly introduced before in Gräfe *et al*. by malachite green based ATPase assays^41^. A reaction velocity v_max_ of 17.1 ± 0.1 nmol min^−1^ per mg purified AtABCG1 and a K_m_ value of 1.8 ± 0.1 mM in comparison to the ATPase hydrolysis deficient EQ/HA mutant were obtained^46,47^. Subsequently, it was demonstrated here for the first time by a functional assay using purified AtABCG1, that the ATPase activity was stimulated by a defined subset of suberin precursor molecules. So far, only few plant ABC transporters, such as AtABCD1^52^, AtABCB25^53^ and ABCG1 from *Nicotiana tabacum*^54^ have been similarly analyzed and shown to be stimulated by their substrates. Here, specific activity of AtABCG1 was stimulated up to 90% by fatty alcohols and acids with chain length of and C_26_–C_30_ and C_24_–C_30_, respectively. Here, the stimulatory effect increased in a chain length dependent manner up to 90% for C_28_ fatty alcohol and 80% for C_26_ fatty acid, respectively and decreased thereafter. Similarly, the *atabcg1* mutant plants exhibited a chain-length dependent decreased relative abundance of C_22_ and C_24_ α, ω- dicarboxylic acids, as well as C_24_ and C_26_ fatty acids and alcohols, respectively. In general, studies with purified protein used in, for example, ATPase assays have to be considered as a more direct approach since a gene knock-out could lead also to artefactual results due to pleiotropic effects^51^. However, the results of the ATPase assay obtained in this study corroborate the mutant studies and simultaneously support the hypothesis, that AtABCG1 is a suberin precursor transporter. Thus, we were able to identify C_22_–C_24_ α, ω- dicarboxylic acids by mutant plant studies as putative substrates and C_26_ fatty alcohols, C_24_ and C_26_ fatty acids by both, direct functional and mutant plant studies, as potential AtABCG1 substrates. In *Arabidopsis*, several half-size transporters were found to be part of suberin formation, but the substrate specificity is different from AtABCG1. AtABCG2, AtABCG6, and AtABCG20 were suspected to transport C_20_ and C_22_ fatty acids, C_22_ fatty alcohol, and C_18:1_ ω-hydroxy fatty alcohols. Single and double mutant plants showed a weaker suberin phenotype than the triple mutant plant^21^. Another half-size transporter, AtABCG11, had a partially similar substrate specificity with decreased abundance of C_20_ fatty acids, C_18_ fatty alcohols, and C_16_ and C_18_ ω-hydroxy fatty acids in the root suberin^23,26^. Hence, suberin deposition may not only depend on one specific transporter, but on the interplay of several half-size ABCG transporters, possibly also by heterodimerization of these transporter proteins. Alternatively, it is conceivable that transporters with overlapping substrate identities could substitute in principle the function of a knocked-out transporter to a certain extent. In accordance, Yadav *et al*. also reported that the expression level of AtABCG16 was significantly increased in *atabcg2 atabcg6 atabcg20* triple mutant plants, although AtABCG16 was so far not shown to be part of suberin formation^21^. Nevertheless, similarly to AtABCG1, a chain-length dependent transport of suberin precursors was proposed for StABCG1 from potato. *StABCG1*-RNAi lines, besides decreased levels of α, ω- dicarboxylic acids, fatty alcohols, and fatty acids, also contained decreased amounts of ω-hydroxyacids with a chain length of C_24_ and longer^34^. Unlike in *Arabidopsis*, downregulation of ABCG1 in *Solanum tuberosum* caused strongly decreased levels of C_18:1_ ω-hydroxyacid and C_18:1_ α, ω- dicarboxylic acid. Moreover, significantly increased amounts of C_16_–C_22_ of ω-hydroxyacids, α, ω- dicarboxylic acids and fatty acids were observed^34^. Despite these differences, the potential StABCG1 substrates, fatty acids and alcohols with >C_26_, also stimulated the specific activity of purified AtABCG1; although they were not detected in *Arabidopsis* suberin so far^34,45^. Compared to previous studies where fatty acids with maximal chain-length of C_24_ and fatty alcohols with maximal chain-length of C_22_ were detected, allowed our method the detection up to even C_26_ fatty alcohols and acids^34,45^. However, this emphasizes that AtABCG1 and StABCG1 exhibit not only sequence similarity, but also a partially overlapping substrate specificity with high affinity for fatty acids and fatty alcohols composed of longer carbon chains. An almost consistent substrate specificity for lipid-like compounds was found for several half-size ABCG transporters, even across different plant species. For instance, ABCG5 from *Oryza sativa* (*O. sativa*) was suggested to transport very-long-chain fatty acids (≥C_28_) and diacids (C_16_) of root suberin monomers^50^ and ABCG1 from *Gossypium hirsutum* (*G. hirsutum*) was found to be part of lipid transport besides cotton fiber development^55^. In *Arabidopsis*, ABCG2, ABCG6, ABCG20, and ABCG11 were shown to be part of suberin barrier formation^21,26,29^, ABCG1, ABCG16, ABCG26, and ABCG9 to be part of pollen protection and development^15–22^, and finally ABCG11, ABCG12, and ABCG13 were found to be part of wax and cutin transport^23–28,56^. Therefore, transport of lipid-like compounds seems to be a well conserved function amongst plant half-size ABCG transporters.

The present study introduces for first time, purification, characterization on enzymatic level, and direct functional assays of a *P. pastoris* expressed plant ABC transporter. Moreover, both approaches, biochemical assays as wells as root suberin analyses indicate the involvement of AtABCG1 in transport of aliphatic suberin precursors with higher chain length (C_24_ or C_26_) in *Arabidopsis* roots.

## Supplementary information

Supplementary Information
