# Supplementary material for: ABCG1 contributes to suberin formation in Arabidopsis thaliana roots
Source: Sci Rep. 2019 Aug 6;9:11381. doi: 10.1038/s41598-019-47916-9 (PMC6684660; doi:10.1038/s41598-019-47916-9)
Supplement: Supplementary file 1 — Supplementary Information [file 41598_2019_47916_MOESM1_ESM.pdf]

Supplementary Information

ABCG1 contributes to suberin formation in

Arabidopsis thaliana roots

Kalpana Shanmugarajah[1,4], Nicole Linka[2], Katharina Gräfe[1,4], Sander H.J. Smits[1], Andreas P.M. Weber[2,4], Jürgen Zeier[3,4] and Lutz Schmitt[1,4]*

[1]Institute of Biochemistry, Heinrich-Heine University, Düsseldorf, Germany

[2]Institute of Plant Biochemistry, Heinrich-Heine University Düsseldorf, Germany

[3]Institute for Molecular Ecophysiology of Plants, Heinrich-Heine University, Düsseldorf, Germany

[4]Cluster of Excellence on Plant Sciences (CEPLAS), Heinrich-Heine University, Düsseldorf, Germany

[*] For correspondence

lutz.schmitt@hhu.de

```
PhABCG1     ------------------------------------------------------------
StABCG1     MSRIVAENMLQGGENVQFYDQRVQQAMEMSQASAYSSPTLGQMLKRVGDVRKEVTGDETP
AtABCG1     MARIV--------------AANDDDSMELNTISSIHDSTLGQLLKNVSDVRKMAIGDETP

PhABCG1     ----------------------------MTNQLPNKVELAEVVPQNGGVFLTWEDLWVTA
StABCG1     VHRILDMSDTQSISSHSLPFVLSFNNLTYSVKVRRKMSFPAILRQPAAGVSTG--DPVAG
AtABCG1     VHESLNQDYND-GYMRTVPFVLSFDNLTYNVSVRPKLDFRNLFPRR----RTE--DPEIA
                                         . .:  *:.:  :. :      *       .

                                       Walker A
PhABCG1     SSVKDGSKAILKGLTGYAMPGELLAIMGPSGSGKSTLLDTIAGRLGSSTRQSGDILINGR
StABCG1     ENLFTNTKFLLNNISGEARDGEIVAVLGASGSGKSTLIDALANRIAKESL-KGTITLNGE
AtABCG1     QTARPKTKTLLNNISGETRDGEIMAVLGASGSGKSTLIDALANRIAKGSL-KGTVKLNGE
            ..    :* :*:.::* :  **::*::* ********:*::*.*:.. :  .* : :**.

                           Q-Loop
PhABCG1     R--QTLAYGSSAYVTQDDTLLATLTIKEAVYYSAELQLPNSMSKSEKKEIADVTLKGMGL
StABCG1     PLDSRLLKVISAYVMQDDLLYPMLTVEETLMFAAEFRLPRSLSKSKKKMRVQALIDQLGL
AtABCG1     TLQSRMLKVISAYVMQDDLLFPMLTVEETLMFAAEFRLPRSLPKSKKKLRVQALIDQLGI
               . :    **** *** *   **::*:: ::**::**.*: **:**  .:. :. :*:

                        ABC Signature              Walker B
PhABCG1     QDAMETRIGGWSGKGISGGQKRRVSICLEILTRPKLLFLDEPTSGLDSAASYYVMKAIAS
StABCG1     RNAAKTIIGDEGHRGVSGGERRRVSIGIDIIHDPILLFLDEPTSGLDSTSAYMVVKVLQR
AtABCG1     RNAAKTIIGDEGHRGISGGERRRVSIGIDIIHDPILLFLDEPTSGLDSTSAFMVVKVLKR
            ::* :* **. . :*:***::***** ::*:  * :************:::: *:*.:

                     H-Loop
PhABCG1     QCQ-GRTIIASIHQPSVDVFSLFHSLCLLSSGRTVYFGPASAANEFFALSGFPCPTLQNP
StABCG1     IAQSGSIVIMSIHQPSYRILGLLDRMLFLSRGQTVYSGSPMNLPHFFSDFGHPIPDSENR
AtABCG1     IAQSGSIVIMSIHQPSHRVLGLLDRLIFLSRGHTVYSGSPASLPRFFTEFGSPIPENENR
             .* *  :* ******  ::.*:. : :** *:*** *      .**:  * * *  :*

PhABCG1     SDHFLKTINSDFDQDIEEGSTRRKSTEEVIDILIKSYKASDKYNAVQSQVA---------
StABCG1     TEFALDLIRE-----LEGSPGGTKSLVEF----NKTWENTKRSNE-NPEIQ---TPTHGL
AtABCG1     TEFALDLIRE-----LEGSAGGTRGLIEF----NKKWQEMKKQSNRQPPLTPPSSPYPNL
            ::. *. *..     :* .    :.  *.     *.::  .: .  :  :

PhABCG1     ------EICQQEGEM-------------------LDKRSHASFITQSLVLTRRSFINMSR
StABCG1     SLKEAISASISRGKLVSGTTSDIHTS-----PASMVPTYANPFWIEMLVLSKRSFTNSWR
AtABCG1     TLKEAIAASISRGKLVSGGESVAHGGATTNTTTLAVPAFANPMWIEIKTLSKRSMLNSRR
                    . ..*::                           :  :  .*::**: *  *

PhABCG1     DLGYYWLRLAVYVVIAVGLGSLYYDVGFSAASVQARGSMLMFVASFITFMAIGGFPSFVE
StABCG1     VPELFGIRLGAIVVTGFILATMFWQLDDSPKGVQERLGFFAFAMSTTFYTCADALPVFLQ
AtABCG1     QPELFGIRIASVVITGFILATVFWRLDNSPKGVQERLGFFAFAMSTMFYTCADALPVFLQ
                : :*:.  *: .. *.:::: :. *  .** * .:: *. *   : . ..:* *::

PhABCG1     DMKVFQREKLNGHYGSGSFVIANTLSAMPYLLLVSLIPGAIAYFMTGLQNGFEHFIYFAL
StABCG1     ERYIFMRETAYNAYRRSSYCLSHAIVSLPALIFLSFAFAAITFWAVGLVGGFSGFLFYFA
AtABCG1     ERYIFMRETAYNAYRRSSYVLSHAIVSFPSLIFLSVAFAATTYWAVGLDGGLTGLLFYCL
            :  :* **.  . *  .*: ::::: ::* *:::*.  .* ::: .** .*:  ::::

PhABCG1     VLFTCMMIVESLMMIVASMVPNFLMGLIAGAGIQALMLLSGGFFRLPNDLPKPFWKYPLH
StABCG1     IILASFWAGNSFVTFLSGVVPSVMLGYTIVVAILAYFLLFSGFFINRDRIP-PYWIW-FH
AtABCG1     IILASFWSGSSFVTFLSGVVPSVMLGYTIVVAILAYFLLFSGFFINRNRIP-DYWIW-FH
            ::::.:   .*:: :::.:**..::*    ..* * :** .***   : :*  :* : :*

PhABCG1     YVAFHKYAYEGMFKNEFEGLKIHDVNGEDILRNTW-------------------------
StABCG1     YLSLVKYPYEAVLQNEFDDATKCFVKGIQLFDNSPLGNVPNALKEKLLSTMSNTLNVKIT
AtABCG1     YMSLVKYPYEAVLQNEFSDATKCFVRGVQIFDNTPLGELPEVMKLKLLGTVSKSLGVTIS
            *::: ** **.:::***.. .   *.* ::: *:

PhABCG1     --------------QMNMDYSKWIDLVILLGMLVLYRVLFLLVVKAGEIVKPAIRAFMSH
StABCG1     SSTCVTTGADILVQQGITDLSKWNCLWITIAWGFFFRVLFYFSLLLGSKNKRR-------
AtABCG1     STTCLTTGSDILRQQGVVQLSKWNCLFITVAFGFFFRILFYFTLLLGSKNKRR-------
                          *   : ***  * * :.  .::*:** : :  *.  *

PhABCG1     SPNQINSAERPLDVFDS
StABCG1     -----------------
AtABCG1     -----------------
```

Figure S 1: Sequence alignment of ABCG1 from *A. thaliana*, *S. tuberosum* and *P. hybrida*. A sequence alignment of AtABCG1 with StABCG1 and PhABCG1 was created using the CLUSTALW program with default settings. The NBD (nucleotide binding domains) is highlighted in blue and the TMD (transmembrane domain) in orange. Conserved amino acids are highlighted in red and conserved motifs marked with a box. Sequence identity of AtABCG1 and StABCG1: 74.15%. Sequence identity of AtABCG1 and PhABCG1: 30.63%. Sequence identity of StABCG1 and PhABCG1: 31.82%.

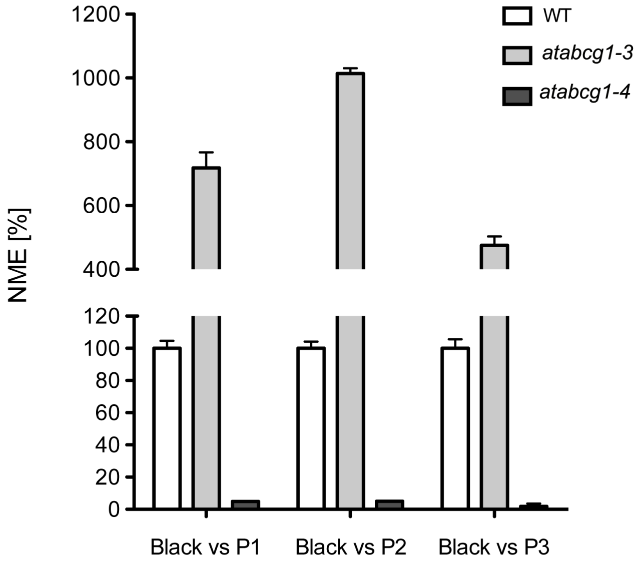

Figure S 2: qRT-PCR of T-DNA insertional *atabcg1* mutant lines. Relative mRNA transcript level of AtABCG1 in *atabcg1-3* and *atabcg1-4* was analyzed by qRT-PCR. P1-P3, primer pairs1-3 bind downstream of the T-DNA; Black, Type 2A phosphatase interacting protein 41 (TIP41)-like reference gene (At4g34270) [1]. The *AtABCG1* transcript-level in *atabcg1-3* is decreased in comparison to the wild type (WT) up to 95 %, while *atabcg1-4* shows a 5- to 10-fold increase. The increased transcript-level in *atabcg1-4* is likely due to a inside the T-DNA region located promotor that causes a strong expression of the 3’ end of the *AtABCG1* mRNA. A functional protein cannot be produced due to the interruption of the coding region by T-DNA [2].

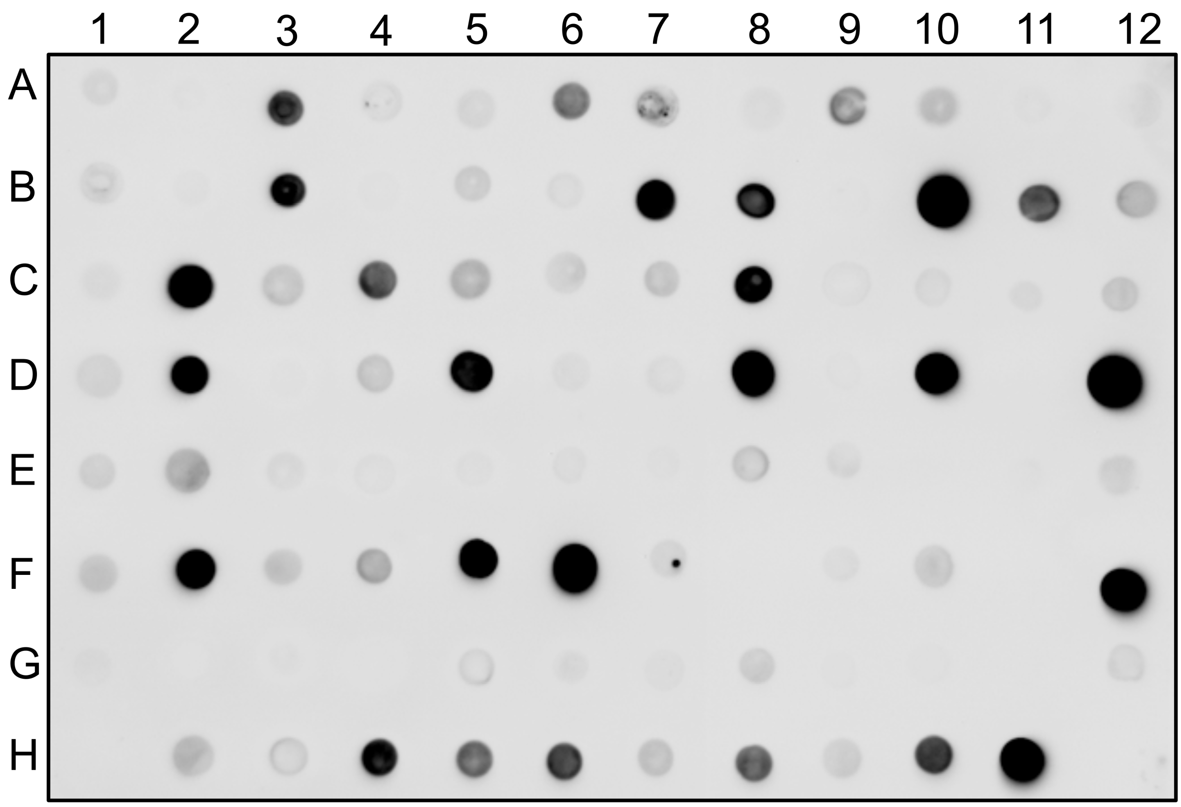

Figure S 3: Solubilization screen of AtABCG1 using Dot Blot technique. AtABCG1 containing *P. pastoris* crude membranes were solubilized with 95 different detergents. The solubilized protein was spotted on a nitrocellulose membrane and analyzed by subsequent immunoblotting (anti-His-tag antibody) for solubilization efficiency. List of used detergents and concentrations can be found in Table S 3. The color of the immunoblot was inverted without contrast change.

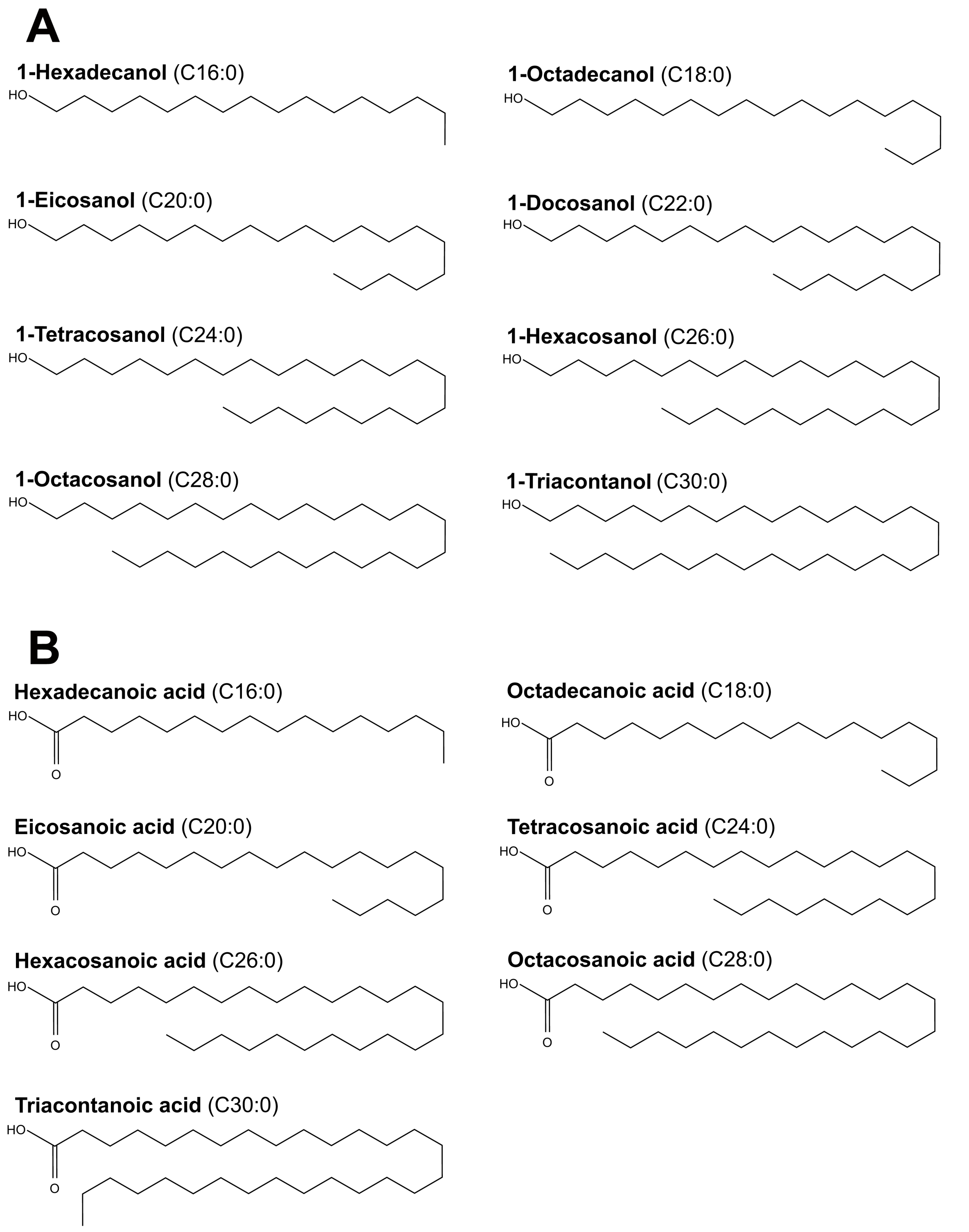

Figure S 4: Structures of substrates used in this study. *A*, fatty alcohols with chain length of C 16:0 to C 30:0. *B*, fatty acids with chain length of C 16:0 to C 20:0 and C 24:0 to C 30:0. Created with Chemdraw 16.0.

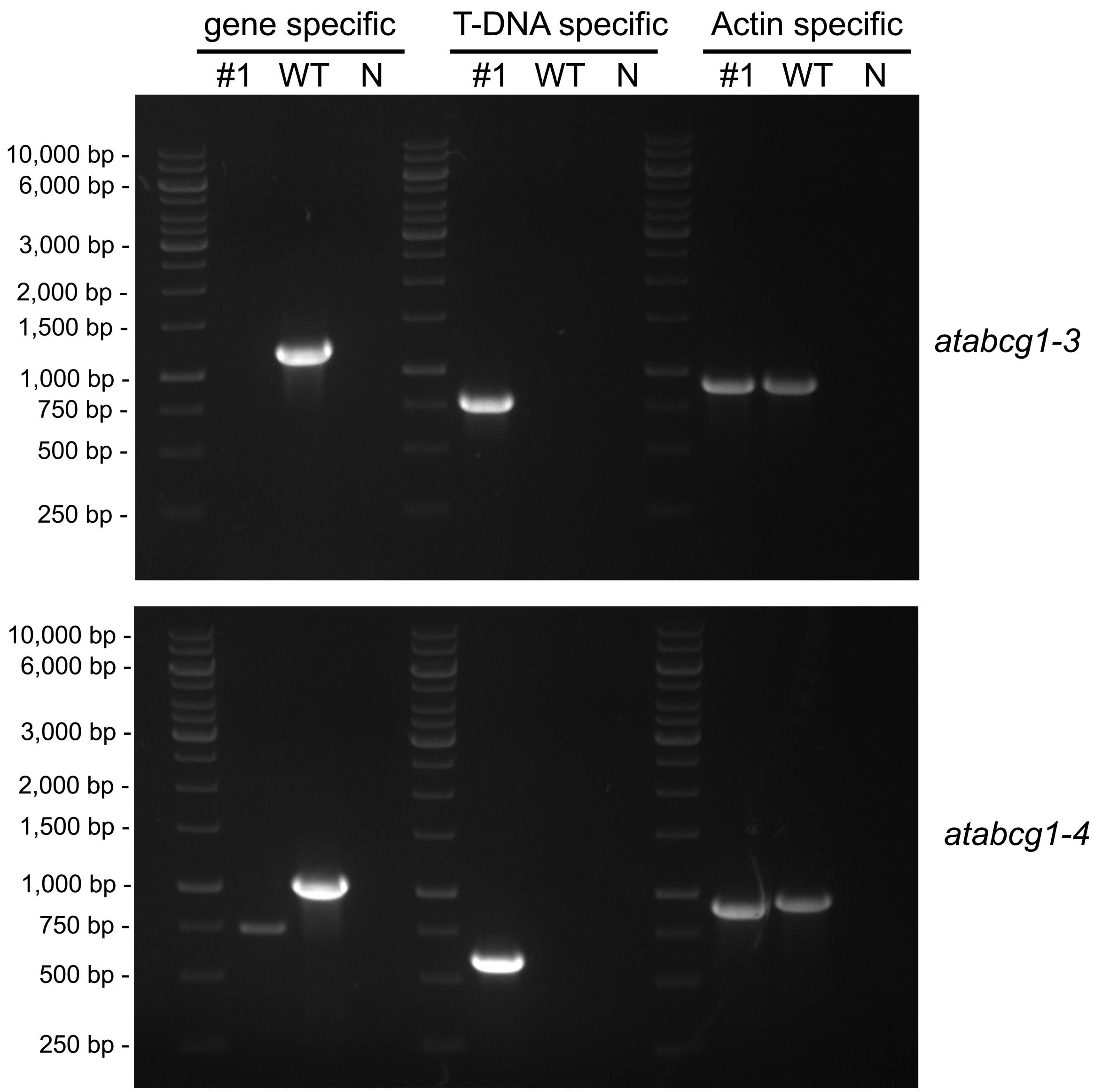

Figure S 5: Genetic analysis of the *atabcg1* mutant lines *atabcg1-3* and *atabcg1-4*. Genomic DNA of wildtype (WT) and mutant lines (#1) were analyzed for *AtABCG1* gene specific, T-DNA specific and actin specific amplification products. N, negative control.

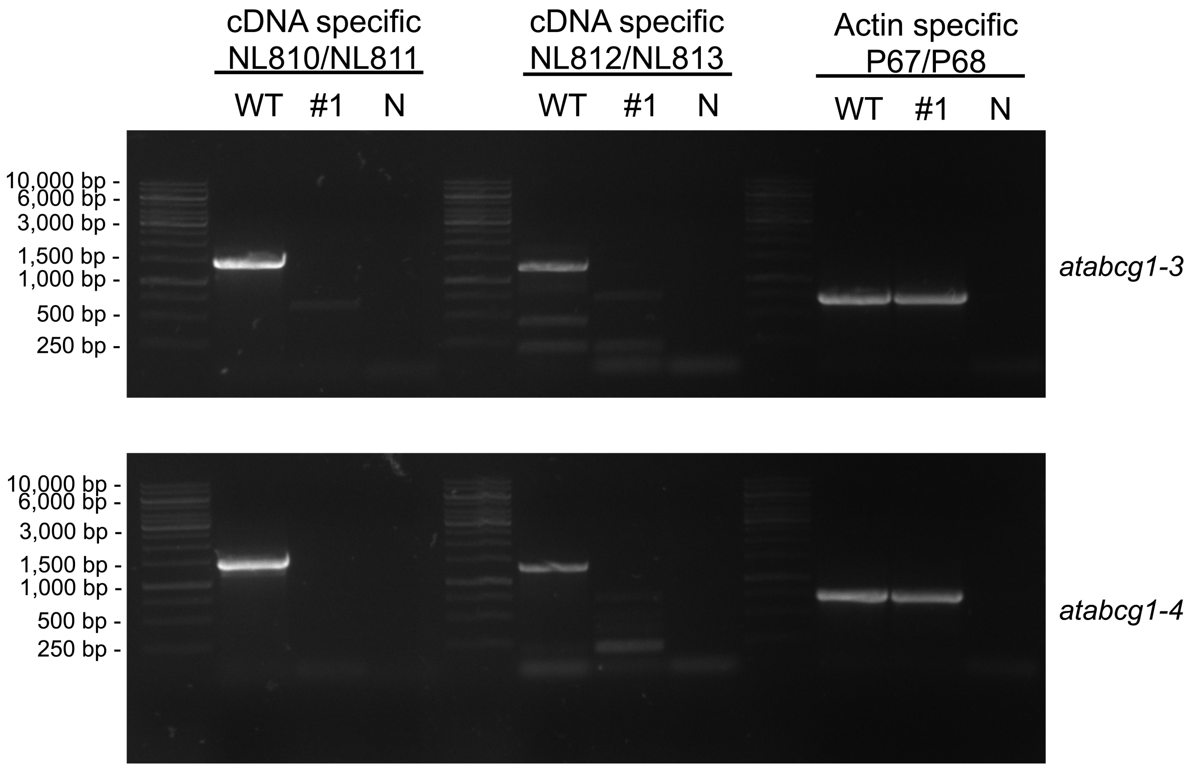

Figure S 6: Presence of full-length AtABCG1 in T-DNA insertional *atabcg1* mutant lines. Amplification of full-length AtABCG1 was performed from cDNA isolated from *atabcg1-3* and *atabcg1-4* lines. WT, wildtype; #1, mutant lines; N, negative control. See Table S 1 for the used primer pairs NL810/NL811, NL812/NL813 and P67/P68.

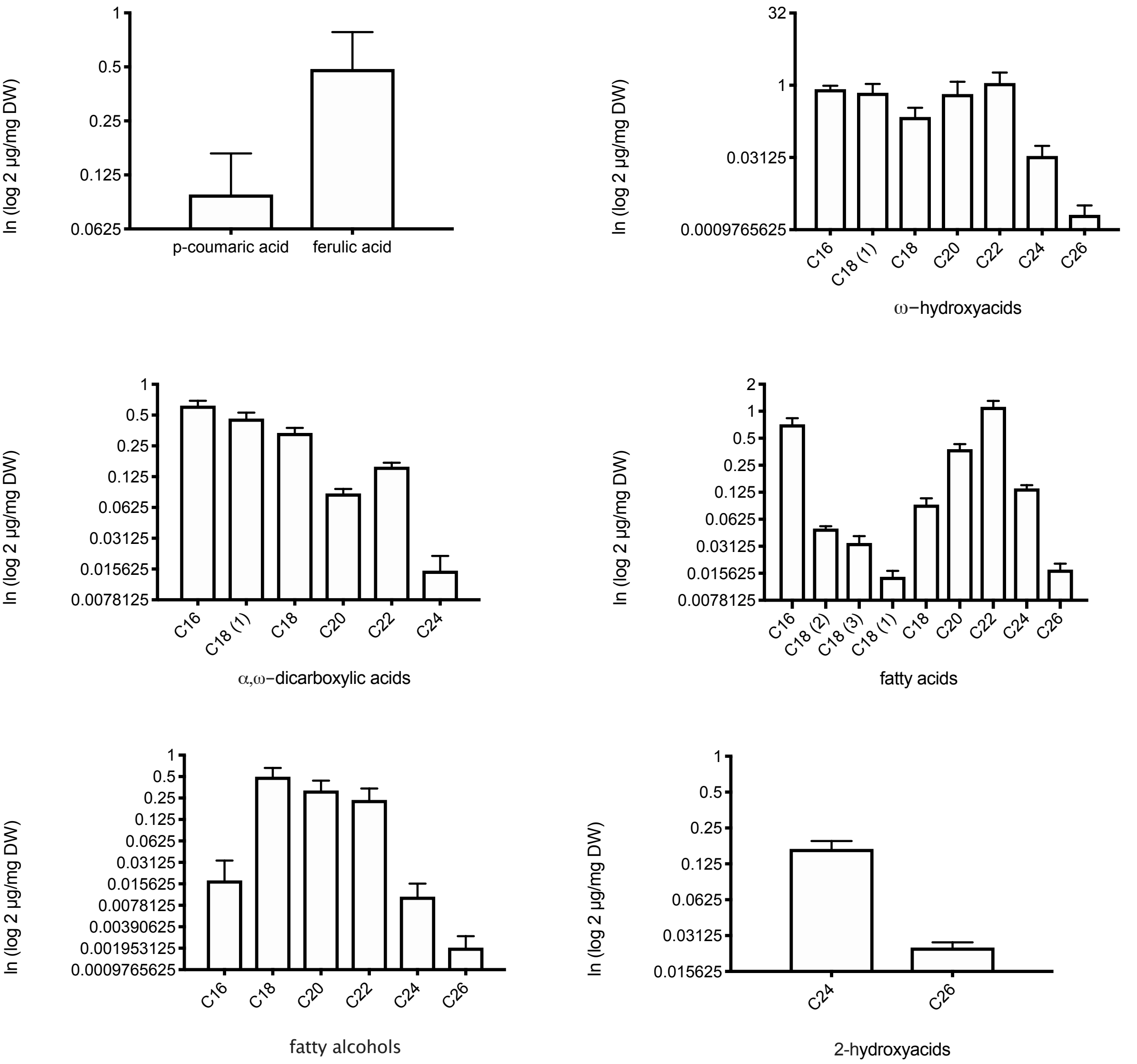

Figure S 7: Suberin composition in *Arabidopsis* wildtype plants. The suberin monomers were quantified using quantifier ions and retention times from Supplemental Table S 2. Data represents results of n=4 independent experiments.

Table S 1: Primers used in this study.

Primer	Application	DNA sequence (5' → 3')
ABCG1-EQ fwd	E259Q mutation	GTTGTTCTTGGACCAGCCAACTTCCGG
ABCG1-EQ rev	E259Q mutation	CCGGAAGTTGGCTGGTCCAAGAACAAC
ABCG1-HA fwd	H291A mutation	GTTATCATGTCCATTGCCCAACCATCCCACAG
ABCG1-HA-rev	H291A mutation	CTGTGGGATGGTTGGGCAATGGACATGATAAC
KS3	SAIL563B03	GCATTGATCGTTTCGAGAGAG
KS4	SAIL563B03	CGAGACTCCAGTTCACGAGTC
KS11	GK850E07	GGATCCGGAGATAGCTCAAAC
KS12	GK850E07	GCATTGATCGTTTCGAGAGAG
P49	LB SAIL line	TAGCATCTGAATTTCATAACCAATCTCGATACAC
P52	LB GK line	CCCATTTGGACGTGAATGTAGACAC
P67	Actin2 fwd	TTCAATGTCCCTGCCATGTA
P68	Actin2 rev	TGAACAATCGATGGACCTGA
QP1	ABCG1_fwd1	GACAACACGCCATTAGGGGA
QP2	ABCG1_rev1	ATCGTTACCCCGAGGGACTT
QP3	ABCG1_fwd2	ACACGCCATTAGGGGAACTAC
QP4	ABCG1_rev2	GAGGGACTTGCTCACTGTACC
QP7	ABCG1_fwd3	AGGTGTGGTTCAGCTGAGTA
QP8	ABCG1_rev3	AAGAACCCAAAAGCAACTGTGA
P90	Black_fwd	GTGAAAACTGTTGGAGAGAAGCAA
P91	Black_rev	TCAACTGGATACCCTTTCGCA
NL810	ABCG1 cDNA fwd2	TCAGAAACGCGGCTAAAACT
NL811	ABCG1 cDNA rev2	ACACCTTGCTGCCTCAGAAT
NL812	ABCG1 cDNA fwd3	CATGCGTACAGTTCCATTCG
NL813	ABCG1 cDNA rev3	GAAGGCGAAGAACCCTAACC

Table S 2: Analytical data of compounds monomers detected by GC/MS.

compound	quantifier ion (m/z)	characteristic ions (m/z)	retention time (min)
p-coumaric acid	250	235, 219, 203	17.7
ferulic acid	250	280, 265, 219	20.5
**ω-hydroxyacids**			
C16	311	343, 159, 146, 103, 75	26.8
C18:1	337	384, 369, 353, 159, 146	29.4
C18	339	371, 159, 146, 103, 75	29.9
C20	367	399, 159, 146, 103, 75	32.9
C22	395	427, 159, 146, 103, 75	35.6
C24	423	455, 159, 146, 103, 75	38.2
C26	451	483, 159, 146, 103, 75	40.6
**α, ω-dicarboxylic acids**			
C16	98	283, 112, 84	25.9
C18:1	98	340, 308, 276	28.6
C18	98	311, 112, 84	29.4
C20	98	339, 112, 84	32.2
C22	98	367, 112, 84	35.1
C24	98	395, 112, 84	37.7
**fatty acid**			
C16	87	270, 74, 143	20.5
C18:2	87	294, 263, 109, 95, 81, 67	23.3
C18:3	87	292, 236,108, 95, 79, 67	23.4
C18:1	87	296, 264, 222, 97, 83	23.5
C18	87	298, 74, 143	24.1
C20	87	326, 74, 143	27.4
C22	87	354, 74, 143	30.6
C24	87	382, 74, 143	33.5
C26	87	410, 74, 143	36.2
**fatty alcohol**			
C16	299	103, 75	21.5
C18	327	103, 75	25.0
C20	355	103, 75	28.2
C22	383	103, 75	31.3
C24	411	103, 75	34.1
C26	439	103, 75	36.8
**2-hydroxyacids**			
C24_2OH	411	455	36.2
C26_2OH	439	483	38.7
**internal standard**			
1-pentadecanol	285	103, 75	19.7
heptadecanoic acid	87	284, 84, 143	22.3

15-hydroxypentadecanoic acid	297	329, 159, 146, 103, 75	25.1

Table S 3: List of used detergents in solubilization screen. A, anionic; Z, zwitterionic; N, nonionic.

Position	Detergent	cmc	used cmc (%)	nature
**A1**	Big CHAP	0.25	1%	N
**A2**	Big CHAP deoxy	0.12	1%	N
**A3**	Anzergent® 3-10	1.2	2%	Z
**A4**	Anameg®-7	0.65	1%	N
**A5**	Anapoe®-C10E6	0.025	1%	N
**A6**	Deoxycholic acid. sodium salt	0.24	1%	A
**A7**	CYGLU®-3	0.86	2%	N
**A8**	Anapoe®-C13E8	0.0055	1%	N
**A9**	Anapoe®-58	0.00045	1%	N
**A10**	Anapoe®-80	0.0016	1%	N
**A11**	2-propyl-1-pentyl maltose	1.9	2%	N
**A12**	Anapoe®-X-405	0.16	1%	N
**B1**	CYMAL®-1	15	2%	N
**B2**	CYMAL®-2	5.4	2%	N
**B3**	Fos-Choline®-12	0.047	1%	Z
**B4**	Anapoe®-X-100	0.015	1%	N
**B5**	Sodium cholate	0.41	2%	A
**B6**	Cyclofos™-2	7.5	1%	Z
**B7**	Anzergent® 3-14	0.007	1%	Z
**B8**	Fos-Choline®-9	1.2	2%	Z
**B9**	Fos-Choline®-Iso-11-6U	0.87	2%	Z
**B10**	Fos-Choline®-16	0.00053	1%	Z
**B11**	Cyclofos™-4	0.45	2%	Z
**B12**	Anapoe®-C10E9	0.053	1%	N
**C1**	Anapoe®-C12E8	0.0048	1%	N
**C2**	Anapoe®-35	0.001	1%	N
**C3**	Anapoe®-C12E9	0.003	1%	N
**C4**	Cyclofos™-6	0.094	1%	Z
**C5**	Anapoe®-C12E10	0.2	1%	N
**C6**	Anapoe®-X-114	0.011	1%	N
**C7**	n-Nonyl-β-D-thiomaltoside	0.15	1%	N
**C8**	Cyclofos™-5	0.15	1%	Z
**C9**	Anapoe®-X-305	-	1%	N
**C10**	CYMAL®-3	0.37	1%	N

**C11**	n-Tetradecyl-N.N-dimethylamine-N-oxide	0.0075	1%	Z
**C12**	Cyclofos™-3	1.3	2%	Z
**D1**	Fos-Choline®-8	3.4	2%	Z
**D2**	Fos-Choline®-Unisat-11-10	0.21	1%	Z
**D3**	2.6-Dimethyl-4-heptyl-β-D-maltose	1.2	2%	N
**D4**	Sucrose monododecanoate	0.016	1%	N
**D5**	n-Dodecyl-α-D-maltoside	0.0076	1%	N
**D6**	CYGLU®-4	0.058	2%	N
**D7**	Dimethyldecylphosphine oxide	0.1	1%	N
**D8**	n-Dodecyl-N.N-dimethylglycine	0.041	1%	Z
**D9**	Fos-Choline®-Iso-11	0.9	2%	Z
**D10**	Fos-Choline®-14	0.0046	1%	Z
**D11**	Fos-Choline®-Iso-9	0.99	2%	Z
**D12**	Fos-Choline®-15	0.0027	1%	Z
**E1**	Tripglu	3.6	2%	N
**E2**	2-Carboxy-5-pentadecenamidopropyldimethylamine	-	1%	Z
**E3**	Fos-Choline®-15	0.0027	1%	Z
**E4**	CYMAL®-5	0.12	2%	N
**E5**	n-Dodecyl-N.N-dimethylamine-N-oxide	0.023	1%	Z
**E6**	CHAPS	0.49	2%	Z
**E7**	MEGA-10	0.21	2%	N
**E8**	n-Octyl-β-D-thiomaltoside	0.4	2%	N
**E9**	Decyl-β-D-glucoside	0.07	1%	N
**E10**	CYMAL®-5	0.12	2%	N
**E11**	n-Undecyl-β-D-thiomaltoside	0.011	1%	N
**E12**	Anapoe®-20	0.0072	1%	N
**F1**	Tetraethylene glycol monoethylether(C8E4)	0.25	1%	N
**F2**	Fosfen™-9	0.014	1%	Z
**F3**	n-Tetradecyl-N.N-dimethylamine-N-oxide	0.0075	1%	Z
**F4**	C-DODECAFOS™	0.77	2%	Z
**F5**	2-Carboxy-w-heptadecenamidopropyldimethylamine	-	1%	Z
**F6**	SDS		10%	
**F7**	n-Heptyl-β-D-thioglucoside	0.85	2%	N
**F8**	Negative control			
**F9**	n-Decyl-α-D-maltoside	-	1%	N
**F10**	LAPAO	0.052	2%	Z
**F11**	PMAL™-8	-	1%	Z
**F12**	n-Dodecyl-β-iminodipropionic acid. monosodium salt	N/A	1%	A

**G1**	Nopol-Fos™	1.4	2%	Z
**G2**	Tripao	4.5	2%	Z
**G3**	MEGA-8	2.5	2%	N
**G4**	PMAL™-C10	-	1%	Z
**G5**	N.N dimethyl(3-carboy-4-dodec-5-ene) aminopropylamine	0.0178	1%	Z
**G6**	Cyclohexyl-n-hexyl-β-D-maltoside	-	1%	N
**G7**	Fosmea®-10	0.15	1%	A
**G8**	n-Dodecyl-β-D-maltoside	0.0087	1%	N
**G9**	NP40	0.05-0.3	1%	N
**G10**	n-Dodecyl-β-D-maltoside	0.0087	1%	N
**G11**	CHAPSO	0.5	2%	Z
**G12**	n-Octyl-β-D-glucoside	0.53	2%	N
**H1**	Decyltrimethylammonium chloride	0.07	1%	C
**H2**	n-Nonyl-β-D-maltoside	0.28	2%	N
**H3**	n-Octyl-β-D-maltoside	0.89	2%	N
**H4**	n-Tetradecyl-β-D-maltoside	0.00054	1%	N
**H5**	n-Undecyl-α-D-maltoside	0.029	1%	N
**H6**	n-Undecyl-β-D-maltoside	0.029	1%	N
**H7**	n-Nonyl-β-D-glucoside	0.2	1%	N
**H8**	n-Dodecyl-β-D-thiomaltoside	0.0026	1%	N
**H9**	Anzergent®3-12	0.094	1%	Z
**H10**	n-Decyl-N.N-dimethylglycine	0.46	2%	Z
**H11**	Sodium dodecanoyl sarcosine	0.42	2%	A
**H12**	PMAL™-C-12	-	2%	Z
**I1**	Dodecyltrimethylammonium chloride	0.0012	1%	C
**I2**	Hexadecyltrimethylammonium chloride	0.000102	1%	C
**I3**	Tetradecyltrimethylammonium chloride	0.0009	1%	C

1 Czechowski, T., Stitt, M., Altmann, T., Udvardi, M. K. & Scheible, W. R. Genome-wide identification and testing of superior reference genes for transcript normalization in Arabidopsis. *Plant Physiol* **139**, 5-17, doi:10.1104/pp.105.063743 (2005).

2 Wang, Y. H. How effective is T-DNA insertional mutagenesis in Arabidopsis? *Journal of Biochemical Technology* **1.1** (2008).
